# The Latin American Registry: 30 years of scientific development and regional cooperation

**DOI:** 10.5935/1518-0557.20210053

**Published:** 2021

**Authors:** Fernando Zegers Hochschild

**Affiliations:** Emeritus professor, Faculty of Medicine, University Diego Portales, Santiago, Chile; Founder of REDLARA and Director of the Latin American Registry of Assisted Reproduction Founder and senior member, Unit of Reproductive Medicine Clinica las Condes, Santiago, Chile

It is with great joy that today, almost 200 centers from 15 countries can celebrate 30 years of scientific development and regional cooperation in the provision of assisted reproductive technology in Latin America. It was in 1990 that for the first time, 19 centers from 8 countries voluntarily agreed to report the outcome of assisted reproductive technology (ART) to a centralized multinational organization. Clinicians and embryologists of these 19 institutions, were of course a bit reluctant to disclose their private information to a third party, but it was the views and spirit of brave founders that overcame these first doubts and voluntarily agreed to use standardized forms and act as one region. The founding institutions can be found in: https://redlara.com/registro.asp.

It is a pleasure and an honor to mention those who are still active and reporting. From Argentina: CRECER, CIGOR, CER and FECUNDITAS; from Colombia: PROFAMILIA and CONCEPTUM; and from Chile, Clinica las Condes and IDIMI

It was after 5 years (1995) of continuous publishing the regional registry, that a group of embryologists together with the clinical directors from each of the 59 reporting centers from 15 countries, gathered for the first time in Valparaiso, Chile and decided to form the Latin American Network of Assisted Reproduction, today known as REDLARA. The objective we had in mind was to engage a larger number of newly established institutions, strengthen scientific cooperation and facilitate the dissemination of knowledge and expertise among institutions in the region. Latin America was divided in 5 sub-regions and embryologists and clinicians, side by side, elected a regional director, responsible for developing continuous educational programs, hands-on working activities, and other sub regional small group learning activities, in other words, organizing regional cooperation programs so that embodied in a common identity, we could all grow together. This is how the transfer of technology and the establishment of quality control programs were implemented and disseminated regionally with the help of embryologists and clinicians from the US and Europe which contributed in numerous hands-on workshops. This form of cooperation defined by the United Nations as South-South and triangular cooperation (UNOSSC) has been pivotal in the implementation and disseminating of reliable and reproducible methods to alleviate the burden experienced by women and men suffering from infertility in our region.

After the birth of Louis Brown in the UK, it took six and a half years for the first IVF baby to be born in Latin America. However, with the establishment of our regional network, it took only one year to report the first ICSI cases after its original publication by Palermo et al. Similar intervals were necessary for the publication of frozen/thawed embryo transfer (FET), oocyte donation (OD) and for the implementation of pre implantation genetic testing (PGT). In these 30 years, technology has experienced dramatic changes and it is our belief, that the transfer, implementation and dissemination of technology have been vastly facilitated within our network. This, together with a continuous online education program for embryologists and clinicians, has become one of the significant achievements of REDLARA.

It is also worth mentioning that three decades ago, we were collecting data by hand and sending it via FAX to the central office in Santiago, Chile. Today, REDLARA has the only cycle based multinational registry, which means that almost 200 institutions in 15 countries collect cycle-based data via an electronic platform equipped with sophisticated validation programs and capable of providing each institution with a detailed numerical as well as graphic printouts of the outcome of their procedures, the country they represent and the region they belong to. This is without doubt a most valuable external quality control program for each center.

But our knowledge and experience has not remained in Latin America. Since 2015, this sophisticated software and registry experience has been shared first with South Africa and now with the African Network and Registry for Assisted Reproductive Technology (ANARA). In this way our regional South-South cooperation has become intercontinental with immense benefit for both Latin America and Africa; and we look forward to a future when we shall be able to systematically examine the influence of ethnic and cultural differences in the way ART is practiced and their impact in the outcome of a variety of reproductive interventions.

For 30 years, IVF centers in Latin America have been capable of overcoming numerous social and economic difficulties but the leading spirit of unity, cooperation and regional identity has prevailed and constitutes an example to the world. We have proved that by working as an inte- grated region, we are capable of incorporating and disseminating sophisticated technology. Our main task today is to make the advances of science and technology, available to a larger proportion of the population in need. It is inequality in access what carries an extra burden to women and men suffering from infertility. Some countries are leading this new journey with laws providing universal access to ART, and we hope many shall follow; but having robust data is a fundamental input when it comes to developing public policies. Our registry can provide every country with solid information, and it is our regional responsibility to use it for the good of our fellow citizens.

These 30 years of regional development would have not been possible without an efficient partnership with the industry responsible for the development of drugs and equipment used in assisted reproductive technology. Among many partner institutions, the generous support by Serono and Organon in the first 15 years was pivotal in structuring REDLARA. Today, the generous and sustained contribution by Ferring pharma, among other companies, provide continuity and help us look into the future with confidence in the many years to come.

